# CO_2_ laser therapy versus topical imiquimod for the treatment of vulvar high‐grade intraepithelial lesions: A retrospective cohort study

**DOI:** 10.1002/ijgo.70083

**Published:** 2025-03-21

**Authors:** Elizabeth Svoboda, Mélanie Benoit, Chelsea Almadin, Valérie Archer‐Déjoie, Coralie Michaud, Julie Lacaille, Laurence Simard‐Émond, Annick Pina, François Gougeon, Marie‐Hélène Mayrand

**Affiliations:** ^1^ Université de Montréal Hospital Research Centre (CRCHUM) Montreal Quebec Canada; ^2^ Department of Obstetrics and Gynecology Université de Montréal Montreal Quebec Canada; ^3^ Department of Pathology and Cellular Biology Université de Montréal Montreal Quebec Canada

**Keywords:** efficacy, imiquimod, laser, safety, treatment failure, vulvar high‐grade squamous intraepithelial lesions, vulvar intraepithelial neoplasia

## Abstract

**Objectives:**

To compare topical imiquimod with CO_2_ laser therapy for the treatment of a first episode of vulvar high‐grade squamous intraepithelial lesions (vHSIL), to identify serious adverse effects of both treatment modalities, and to examine risk factors for treatment failure.

**Methods:**

This retrospective chart‐based cohort study included 47 patients initially treated with topical imiquimod or CO_2_ laser therapy between 2017 and 2021. The primary outcome was treatment failure, defined as the need for repeat treatment. Cumulative incidence curves were used to compare the probability of treatment failure over time by treatment group. Potential risk factors for treatment failure, including age, treatment type, lesion focality, and smoking, were examined using Cox proportional hazards models to estimate adjusted hazard ratios (aHRs) and 95% confidence intervals (95% CIs).

**Results:**

Sixty‐six percent (31/47) of patients were initially treated with topical imiquimod and 34% (16/47) with CO_2_ laser. Both groups were similar in age, lesion focality, human papillomavirus vaccination, and smoking status. During a follow‐up of 62.3 person‐years, 52% (16/31) of patients treated with imiquimod and 56% (9/16) of patients treated with CO_2_ laser experienced treatment failure. Age over 52 years was associated with a higher risk of treatment failure (aHR 3.07, 95% CI 1.25–7.53). The association was positive but not significant for multifocal versus unifocal lesions and for smokers versus non‐smokers. No serious adverse effects were observed with either treatment modality.

**Conclusion:**

Topical imiquimod has similar efficacy and safety to CO_2_ laser therapy for initial the treatment of vHSIL. Older age is associated with an increased risk of treatment failure.

## 1 INTRODUCTION

Vulvar intraepithelial neoplasia (VIN) is a premalignant disease of the vulva. The current classification of vulvar squamous intraepithelial lesions includes low‐grade squamous intraepithelial lesions (LSIL), high‐grade squamous intraepithelial lesions (HSIL), differentiated type VIN (dVIN) and vulvar aberrant maturation (VAM).^1^ The incidence of vulvar HSILs (vHSIL) has been increasing in recent years, particularly within the population of women aged 35–55 years.^2^, ^3^ vHSIL is associated with human papillomavirus (HPV) infection, particularly genotype 16. Risk factors include smoking, age over 50 years, and immunosuppression.^3^, ^4^ Approximately 10% of vHSIL will progress to invasive carcinoma within 10 years if left untreated.^5^, ^6^

In the past, surgical excision was the treatment of choice, including vulvectomy when lesions were diffuse and multifocal.^4^ Excision has the advantage of providing a specimen for pathology. However, surgical margins are often positive. Achieving negative margins can lead to important anatomical changes with subsequent increased risk of sexual and urinary dysfunction.^7^ In addition to preventing progression to malignancy, important treatment aims now include preservation of anatomy, function, and quality of life.^7^ CO_2_ laser ablation was introduced as a more conservative approach for when cancer is not suspected.^8^ While laser therapy is a relatively straightforward procedure, its availability is limited due to the high cost of equipment and the specialized expertise needed for maintenance.^9^ In addition, due to its mechanism of action, some patients experience significant post‐treatment pain and bothersome pigmentation changes.^10^ More recently, topical imiquimod, an immunomodulating cream, has gained popularity as an off‐label option for the treatment of vHSIL.^11^

The literature comparing the use of topical imiquimod with CO_2_ laser therapy remains limited. A recent randomized controlled trial suggested that topical imiquimod was non‐inferior to surgery, which included excision or laser ablation, or both.^12^ However, the study population included several cases of recurrent HSIL, which limits its generalizability to initial, potentially less complex cases. Other observational studies have shown similar recurrence probabilities between treatment modalities.^13^, ^14^, ^15^, ^16^ Previous studies were mostly limited by small sample sizes or lack of adjustment for potential confounders, or both.^13^, ^14^, ^15^, ^16^

We decided to conduct a retrospective cohort study to compare the probability of treatment failure after initial treatment with either topical imiquimod or CO_2_ laser therapy for a first episode of vHSIL. We also sought to identify serious adverse effects of both treatment modalities and to examine risk factors for treatment failure.

## 2 MATERIALS AND METHODS

### 2.1 Study design and population

A retrospective chart review was performed at the University of Montreal Hospital Center (CHUM), a tertiary hospital in Montreal, Canada. We included all patients who consulted the colposcopy clinic for an initial treatment of vHSIL between June 2017 and July 2021. Patients were eligible if they had a first diagnosis of vHSIL and had not received any treatment before being referred to the CHUM. Other inclusion criteria were that the initial treatment was either topical imiquimod or CO_2_ laser therapy, that the prescribed treatment was used, and that there was at least one follow‐up visit after completion of treatment. The date of inclusion (baseline) in the study was defined as the date of prescription of the initial treatment.

Exclusion criteria included pregnancy, recurrent VIN, dVIN, VAM or p16‐negative intraepithelial lesions at presentation, suspected or confirmed invasive disease, and initial treatment with both topical imiquimod and CO_2_ laser therapy.

**FIGURE 1:**
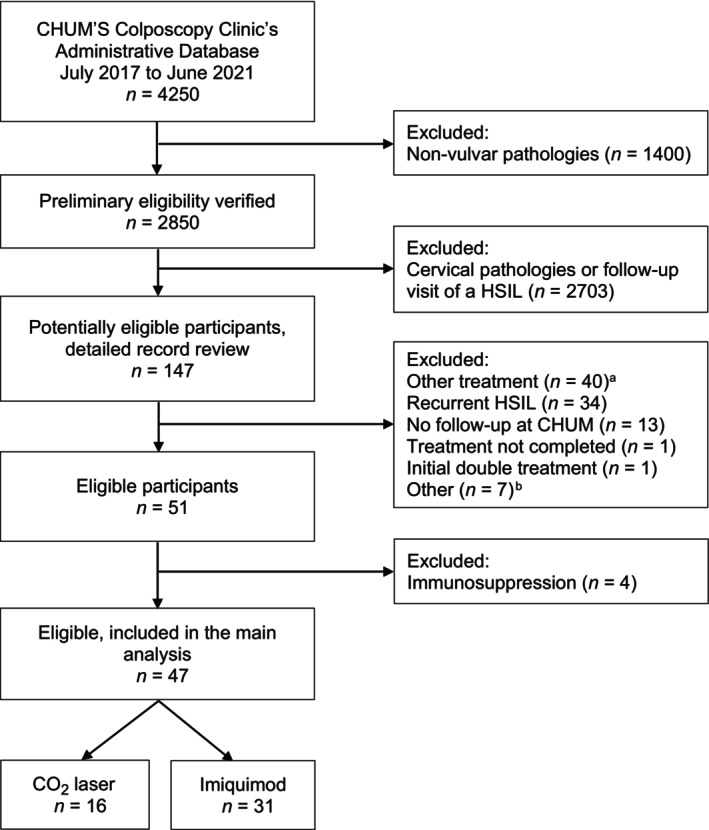
Flowchart for participant selection. ^a^Vulvectomy, loop electrosurgical excision procedure (LEEP), electrocoagulation, cryotherapy. ^b^Pregnancy, suspected/confirmed invasive disease. CHUM, University of Montreal Hospital Center; HSIL, high‐grade squamous intraepithelial lesion.

In total, approximately 4250 potentially eligible patient records were identified for the study period, of which 147 underwent a thorough record review (Figure 1). Of these, 51 patients were eligible. We further excluded four women who were immunosuppressed because they were likely to substantially differ from other participants in their risk of outcome and were too few to enable a stratified analysis. Thus, 47 participants were included in the main analysis. The study was approved by the CHUM Institutional Review Board. Informed consent was waived due to the retrospective nature of the study design.

### 2.2 Data collection

Electronic patient records were reviewed for demographic and clinical information, including age, treatment modality, lesion focality, tobacco use, HPV vaccination, serious adverse effects, and outcome information. Study data were collected and managed using Research Electronic Data Capture (REDCap).

The initial treatment modality was either topical imiquimod or CO_2_ laser therapy. The choice of treatment was based on a shared decision‐making process influenced by patient and healthcare provider preferences. Imiquimod was prescribed at a 5% concentration in increasing doses up to three times per week until lesion clearance for a maximum of 16 weeks. Patients received detailed counseling on potential local and systemic side effects prior to treatment initiation. Patients were seen 4–6 weeks after starting treatment, and then every 6 months. Individualized CO_2_ laser vaporization was conducted in the outpatient clinic. After disinfection, local anesthesia was performed with subcutaneous 1% xylocaine. The Lumenis UltraPulse CO_2_ laser, coupled to a colposcope, was used with a power density of 1000 W/cm^2^. Experienced colposcopists proceeded with laser fulguration under vulvoscopy guidance. Lesions were destroyed to a depth of 2 mm with margins of 3–5 mm of healthy tissue. Patients were seen 6 weeks postoperatively and subsequently every 6 months.

### 2.3 Outcome and follow‐up

The primary outcome was treatment failure, defined as the need for another treatment. Treatment failure was suspected based on vulvar colposcopic examination and confirmed by biopsy. The date of treatment failure was the date that another treatment was prescribed. Survival time in the cohort was calculated from baseline and then until participants were prescribed a different treatment, were lost to follow‐up, completed their last follow‐up visit, or when the study ended in October 2023. The secondary outcome was the occurrence of serious adverse effects, which we defined as the need for an emergency room consultation, hospitalization, use of oral or intravenous antibiotics, or prescription of narcotics for pain relief.

### 2.4 Statistical analysis

Baseline characteristics of participants were described as means and standard deviations for continuous variables, and frequencies and percentages for categorical variables. Comparisons between treatment groups were made using *t*‐tests and Fisher exact tests. Statistical significance was set at a *P*‐value of 0.05. Cumulative incidence curves were generated to visualize the probability of treatment failure over time by treatment group and compared using the log‐rank test. Cox proportional hazards models were used to calculate hazard ratios (HRs) and 95% confidence intervals (95% CIs) for the association between each baseline predictor and vHSIL treatment failure.

Potential predictors of treatment failure were identified through a literature review and included age, treatment modality, lesion focality, tobacco use, and HPV vaccination. Age at treatment prescription was dichotomized based on the median to compare older versus younger age. Lesion focality was classified as unifocal or multifocal. Tobacco use was reported as active smoking, no active smoking, or missing data. Given the low proportion of missing data (<4%) for this variable, we used simple imputation of the mode (i.e., non‐smoker). Finally, HPV vaccination was defined as having received at least one dose of the vaccine or not having received any doses. Of 19 participants with available vaccination status, only one was vaccinated. We imputed missing values as never having been vaccinated, given that most participants were over the age of regulatory approval (45 years), that vaccination coverage is exceedingly low in this age group, and that even with counseling and a prescription given at our clinic, we could find no record of vaccination during the study period. As only one patient was vaccinated, this variable was not included in the models.

Multivariate Cox regression models were adjusted for age, treatment modality, lesion focality, and smoking status. The proportional hazards assumption was assessed using graphics of Schoenfeld residuals and the Grambsch–Therneau test. Based on both approaches, the assumption was satisfied. Statistical analyses were performed using R version 4.4.1 (R Core Team, 2024).

## 3 RESULTS

Of the 47 patients included in this study, 16 (34%) were initially treated with CO_2_ laser and 31 (66%) with topical imiquimod (Table 1). Twenty‐nine (94%) patients in the imiquimod group were prescribed 5% imiquimod per standard protocol. Due to a shortage of 5% imiquimod during the study period, 2 (6%) patients were prescribed 3.75% imiquimod daily. The mean age (SD) at prescription of the initial treatment was 53 (14) years. Just over half (26/47, 55%) of the participants had unifocal lesions. The majority were non‐smokers (27/47, 57%) and had not been vaccinated against HPV (46/47, 98%). Patients in the CO_2_ laser and topical imiquimod groups did not differ significantly with respect to age, lesion focality, HPV vaccination, and active smoking (*P >* 0.05 for all variables).

**TABLE 1:** Baseline characteristics of the study participants.

Characteristics	Overall (*n* = 47)	CO_2_ laser (*n* = 16)	Imiquimod (*n* = 31)	*P*‐value^a^
Age (years)				
Mean (SD)	53.1 (13.6)	53.2 (14.5)	53.1 (13.4)	0.977
≤52^b^	25 (53.2)	10 (62.5)	15 (48.4)	0.538
>52	22 (46.8)	6 (37.5)	16 (51.6)	
HPV vaccination				
No	46 (97.9)	16 (100.0)	30 (96.8)	>0.99
Yes	1 (2.1)	0 (0.0)	1 (3.2)	
Type of lesion				
Unifocal	26 (55.3)	10 (62.5)	16 (51.6)	0.547
Multifocal	21 (44.7)	6 (37.5)	15 (48.4)	
Active smoking				
No	27 (57.4)	8 (50.0)	19 (61.3)	0.541
Yes	20 (42.6)	8 (50.0)	12 (38.7)	

*Note*: Data are presented as mean (SD) for continuous variables and *n* (%) for categorical variables.

^a^ *t*‐tests were used for continuous variables, and Fisher exact tests were used for categorical variables, with a significance level of 0.05.

^b^ Dichotomized based on the median.

During follow‐up (median 7.4 months; interquartile range 4.6–22.0 months), 25 (53%) participants experienced treatment failure, i.e., the need for another treatment. Sixteen (52%) patients in the imiquimod group had a treatment failure at a mean follow‐up of 18.2 months, while 9 (56%) patients in the CO_2_ laser group had a treatment failure at a mean follow‐up of 11.4 months.

**FIGURE 2:**
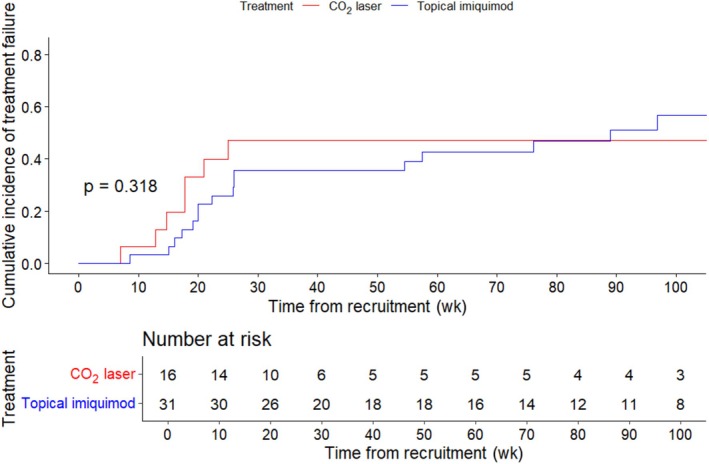
Cumulative incidence curves of treatment failure by treatment modality and *P*‐value of the log‐rank test. Incidence curves were estimated as 1 minus the Kaplan–Meier survival function (no competing risks considered).

Median time to treatment failure was 4.8 months (imiquimod, 5.5 months; CO_2_ laser, 4.1 months). Cumulative incidence curves (Figure 2) suggested that the probability of treatment failure over time was comparable between treatment modalities (*P* = 0.318).

In univariate Cox proportional hazards models (Table 2), age >52 years was associated with a higher risk of treatment failure compared with age ≤52 years. The association was positive for smokers versus non‐smokers (HR 1.42, 95% CI 0.64–3.17), and inverse for topical imiquimod versus CO_2_ laser (HR 0.66, 95% CI 0.29–1.50). The association was virtually null for multifocal versus unifocal lesions. In multivariate Cox proportional hazards models (Table 2), HR values were higher, and the direction of the association was positive for multifocal versus unifocal lesions, although not significant.

**TABLE 2:** Hazard ratios (HRs) and 95% confidence intervals (CIs) for each predictor in relation to vulvar high‐grade squamous intraepithelial lesion (vHSIL) treatment failure.

Predictor	Person‐months of follow‐up	Treatment failures (*N*)	Univariate HR (95% CI)	Multivariate HR^a^ (95% CI)
Treatment				
CO_2_ laser	182.6	9	1.00 (ref.)	1.00 (ref.)
Topical imiquimod	565.5	16	0.66 (0.29–1.50)	0.53 (0.22–1.27)
Age^b^ (years)				
≤52	420.4	9	1.00 (ref.)	1.00 (ref.)
>52	327.7	16	2.41 (1.06–5.47)	3.07 (1.25–7.53)
Type of lesion				
Unifocal	409.6	13	1.00 (ref.)	1.00 (ref.)
Multifocal	338.5	12	1.06 (0.48–2.32)	1.61 (0.68–3.82)
Active smoking				
No	500.9	13	1.00 (ref.)	1.00 (ref.)
Yes	247.2	12	1.42 (0.64–3.17)	1.50 (0.67–3.37)

^a^ Adjusted for all the variables in the table.

^b^ Dichotomized based on the median.

No serious adverse events were observed with either topical imiquimod or CO_2_ laser therapy over the course of the study period.

In a sensitivity analysis including the four patients initially excluded because of immunosuppression (Table S1), the adjusted HRs did not appreciably change. Three of the four (75%) immunosuppressed women experienced treatment failure, with a median time to treatment failure of 2.7 months. The association between the presence (vs absence) of immunosuppression and treatment failure was positive but not significant (aHR 2.76, 95% CI 0.75–10.09).

## 4 DISCUSSION

### 4.1 Principal findings

In this retrospective, chart‐based cohort study of women treated with either topical imiquimod or CO_2_ laser therapy for a first episode of vHSIL, 25 (53%) patients experienced treatment failure. Both treatment modalities showed a comparable risk of treatment failure. Patients in the imiquimod group had a median time to treatment failure of 5.5 months, compared with 4.1 months in the CO_2_ laser group. No serious adverse effects were observed in either treatment group. Age >52 years was positively associated with treatment failure. There was a suggestion of an increased risk of treatment failure with multifocality, active smoking, and immunosuppression.

### 4.2 Results in the context of what is known

Consistent with our study, previous studies have reported vHSIL recurrence probabilities ranging from 20% to 51%.^12^, ^13^, ^14^, ^15^, ^16^ Differences in recurrence probabilities between studies may be partly explained by different treatment regimens and length of follow‐up.^12^, ^13^, ^14^ We found comparable treatment failure probabilities over time between groups, with 52% and 56% observed for the topical imiquimod and CO_2_ laser groups, respectively. The majority of previous studies have reported similar recurrence probabilities between the two treatment modalities.^13^, ^14^, ^15^ Only one retrospective cohort study of 303 patients with vHSIL found a significantly higher proportion of recurrences with CO_2_ laser than with imiquimod.^16^ However, in multivariate models, the risk of recurrence was similar between treatments.^16^ In the present study, imiquimod was associated with a lower, though not statistically significant, risk of treatment failure compared with CO_2_ laser. Similarly, a retrospective cohort study of 73 patients with vHSIL suggested a higher recurrence‐free survival with imiquimod compared with laser ablation.^15^ However, this study was limited by a small sample size, particularly the small number of participants in the imiquimod group (*n* = 6).^15^

In terms of safety, similar results have been reported in the literature, with complications being rare with both modalities.^7^, ^10^, ^17^ Topical imiquimod has limited side effects such as burning or pain.^11^, ^17^, ^18^ Common side effects of CO_2_ laser ablation also include burning, pain, and pruritus.^7^, ^10^, ^17^

We observed that older age, multifocality, and active smoking may adversely affect treatment response. Previous studies have suggested that age >50 years and multifocality are associated with vHSIL recurrence.^3^, ^4^, ^15^, ^19^ However, the results of previous studies on the association between smoking and vHSIL recurrence have been inconclusive.^3^, ^15^, ^16^, ^19^ Smoking reduces the local immune response, which may affect vHSIL treatment outcomes by contributing to HPV persistence.^15^

Sensitivity analysis suggested that immunosuppression may be associated with treatment failure, which is consistent with previous studies.^3^, ^19^ In addition, we observed that immunosuppressed women tended to have a shorter median time to treatment failure, as suggested by a previous study.^15^

### 4.3 Clinical implications

This study suggests that topical imiquimod is a safe and effective alternative to CO_2_ laser therapy for the treatment of a first episode of vHSIL. This finding is particularly important for centers where CO_2_ laser may not be readily available, as topical imiquimod could be prescribed instead, reducing the hassle for patients who must travel to larger centers to receive CO_2_ laser. In addition to availability, a disadvantage of CO_2_ laser therapy is the occasional skin changes including scarring, hypo‐ or hyper‐pigmentation, which can make it difficult to rule out recurrent lesions.^20^

The choice of initial treatment should also be based on patient preference and compliance.^12^ Adherence to topical imiquimod may be lower due to the longer duration of treatment and consequently longer‐lasting side effects.^12^ In this study, one woman (2%) was excluded for not completing imiquimod for the prescribed duration. Differences in treatment duration and, possibly, individual insurance coverage may pose challenges in conducting cost‐effectiveness studies.

### 4.4 Strengths and limitations

Although this study is limited by retrospective data collection, we were able to collect comprehensive demographic and clinical information, including key risk factors for treatment failure. Furthermore, because data were collected from medical records and self‐reported variables were easy to recall (e.g., active smoking), the risk of information bias is likely to be minimal. The proportion of missing data for HPV vaccination was high (60%), but given the average age of the participants, the likelihood of vaccination was very low, so we imputed missing values as never having been vaccinated. The impact of HPV vaccination on vHSIL treatment outcomes should be addressed in future studies. The VIVA (Vaccine to Interrupt Progression of Vulvar and Anal Neoplasia) trial is currently investigating this question and may provide valuable insights.^21^

Selection bias is a potential limitation, as personal preferences of colposcopists may have led to more frequent use of imiquimod for larger lesions, which would have disadvantaged this treatment compared with CO_2_ laser ablation. Patients had to have used the prescribed treatment to be included in the analysis, but only one woman was excluded for this reason (Figure 1).

Another limitation of this study is that in patients treated with imiquimod who had a short follow‐up, we were unable to determine whether discontinuation was due to treatment failure or poor tolerability. Women who started imiquimod with more frequent application (e.g., three times per week) may have experienced more side effects that led to discontinuation.

A key strength of this study is the strict inclusion criteria, which allowed us to include only patients with a first diagnosis of vHSIL and to exclude recurrent VIN, dVIN, and suspected or confirmed invasive disease. The generalizability of the results to a different patient population may be limited because we conducted this study at a tertiary care center. This may mean that less complex patients were treated at their own other centers.

The number of participants in this study is small, although similar to the numbers available in the current literature. Due to the limited sample size, we were unable to determine whether the response to each treatment modality differed by immunosuppression status. Given that topical imiquimod is an immune response modifier, it may be less effective in immunocompromised women.^22^ Furthermore, we lacked information on HPV status. Post‐treatment HPV positivity may be less common in cases with a complete response to imiquimod than in cases with a partial or no response.^23^ Prospective studies with larger sample sizes, ideally randomized, should be conducted to better understand the impact of these factors on treatment outcomes.

## 5 CONCLUSIONS

In this retrospective cohort study, topical imiquimod and CO_2_ laser therapy had comparable risks of treatment failure for a first episode of vHSIL. Both options were safe, with no serious adverse effects reported. Patients older than 52 years had an increased risk of treatment failure.

## AUTHOR CONTRIBUTIONS

**E.S.**: methodology, investigation, data curation, formal analysis, writing—original draft. **M.B.**: methodology, data curation, formal analysis, writing—original draft, visualization. **C.A.**: methodology, writing—review and editing. **V.A.‐D.**: investigation, writing—review and editing. **C.M.**: methodology, writing—review and editing. **J.L.**: project administration, investigation, data curation, writing—review and editing. **L.S.‐É.**: conceptualization, writing—review and editing. **A.P.**: conceptualization, writing—review and editing. **F.G.**: conceptualization, writing—review and editing. **M.‐H.M.**: conceptualization, methodology, data curation, formal analysis, writing—original draft, supervision, project administration. All authors have agreed to the published version of the manuscript.

## FUNDING INFORMATION

The authors have nothing to report.

## CONFLICT OF INTEREST STATEMENT

The authors have no conflicts of interest.

## Supporting information


Data S1.

## Data Availability

The data that support the findings of this study are available from the corresponding author (M.‐H.M.) upon reasonable request.
