# Supplementary material for: CO2 laser therapy versus topical imiquimod for the treatment of vulvar high‐grade intraepithelial lesions: A retrospective cohort study
Source: Int J Gynaecol Obstet. 2025 Mar 21;170(2):901–7. doi: 10.1002/ijgo.70083 (PMC12255928; doi:10.1002/ijgo.70083)
Supplement: Supplementary file 1 — Data S1. [file IJGO-170-901-s001.docx]

**Table S1.** Hazard ratios and 95% confidence intervals for each predictor in relation to vHSIL treatment failure, including immunocompromised women (n=51).

| **Predictor** | **Person-months of follow-up** | **N, Treatment failures** | **Univariate**  **HR (95% CI)** | **Multivariate^a^**  **HR (95% CI)** |
| --- | --- | --- | --- | --- |
| **Treatment** |  |  |  |  |
| CO2 laser | 185.1 | 10 | 1.00 (ref.) | 1.00 (ref.) |
| Topical imiquimod | 614.5 | 18 | 0.63 (0.29 – 1.37) | 0.48 (0.21 – 1.11) |
| **Immunosuppression** |  |  |  |  |
| No | 748.1 | 25 | 1.00 (ref.) | 1.00 (ref.) |
| Yes | 51.5 | 3 | 1.88 (0.57 – 6.28) | 2.76 (0.75 – 10.09) |
| **Age** |  |  |  |  |
| ≤52 years | 414.1 | 11 | 1.00 (ref.) | 1.00 (ref.) |
| >52 years | 385.4 | 17 | 1.78 (0.83 – 3.81) | 2.34 (1.03 – 5.31) |
| **Type of lesion** |  |  |  |  |
| Unifocal | 443.8 | 14 | 1.00 (ref.) | 1.00 (ref.) |
| Multifocal | 355.8 | 14 | 1.16 (0.55 – 2.45) | 1.58 (0.71 – 3.49) |
| **Active smoking** |  |  |  |  |
| No | 552.4 | 16 | 1.00 (ref.) | 1.00 (ref.) |
| Yes | 247.2 | 12 | 1.26 (0.59 – 2.70) | 1.49 (0.66 – 3.35) |
| ^a^Adjusted for all the variables in the table. | | | | |
